# Long-Term Exposure to Cadmium Causes Hepatic Iron Deficiency through the Suppression of Iron-Transport-Related Gene Expression in the Proximal Duodenum

**DOI:** 10.3390/toxics11070641

**Published:** 2023-07-24

**Authors:** Maki Tokumoto, Jin-Yong Lee, Yasuyuki Fujiwara, Masahiko Satoh

**Affiliations:** 1 Laboratory of Pharmaceutical Health Sciences, School of Pharmacy, Aichi Gakuin University, Nagoya 464-8650, Japan; maki@dpc.agu.ac.jp (M.T.); leejy@dpc.agu.ac.jp (J.-Y.L.); 2 Department of Environmental Health, School of Pharmacy, Tokyo University of Pharmacy and Life Sciences, Hachioji 192-0392, Japan; yasuyuki@toyaku.ac.jp

**Keywords:** cadmium, iron deficiency, iron storage, iron-transport-related factor, proximal duodenum

## Abstract

Cadmium (Cd) is an environmental pollutant that damages various tissues. Cd may cause a depletion of iron stores and subsequently an iron deficiency state in the liver. However, the molecular mechanism of decreased iron accumulation in the liver induced by long-term exposure to Cd is unknown. In this study, we investigated the hepatic accumulation of iron and the proximal duodenal expression of the genes involved in iron transport using mice chronically exposed to Cd. Five-week-old female C57BL/6J mice were fed a diet containing 300 ppm Cd for 12, 15, 19 and 21 months. The iron concentration in the liver was markedly decreased by Cd. Among iron-transport-related genes in the proximal duodenum, the gene expression of *HCP1* and *Cybrd1* was significantly decreased by Cd. *HCP1* is an influx transporter of heme iron. *Cybrd1* is a reductase that allows non-heme iron to enter cells. The expression of iron-transport-related genes on the duodenal basolateral membrane side was hardly altered by Cd. These results suggest that long-term exposure to Cd suppresses the expression of *HCP1* and *Cybrd1* in the proximal duodenum, resulting in reduced iron absorption and iron accumulation in the liver.

## 1. Introduction

Cadmium (Cd) is a toxic heavy metal and well-known causative agent of Itai-itai disease, a pollution disease identified in Japan in the 1950s. Cd causes damages in the liver, kidney and bone via oral exposure, and respiratory damage through inhalation exposure [1,2,3]. The main target of chronic exposure to Cd is renal proximal tubules, leading to the development of osteomalacia in Itai-itai disease. Cd is widely present in the environment and accumulates easily in rice and seafood. Therefore, humans are exposed to Cd through their diet, which lasts a lifetime. Additionally, the absorbed Cd is retained in the body for a long time, because Cd has a very long biological half-life of 15–30 years [1]. While Cd causes various chronic toxicities, including renal toxicity, it also causes iron deficiency and iron deficiency anemia. In humans, there is a high correlation between blood Cd concentration and iron deficiency or iron deficiency anemia [4,5,6,7]. Previous studies have also demonstrated that oral exposure to Cd decreases the iron concentration in the liver of mice (10 mg Cd/kg body weight for 14 days [8], 100 and 200 ppm for 85 days [9]), rats (0.6 mg Cd per kg of feed for 30 days [10], 2.5, 5, 10, 25, 50 and 100 ppm Cd for 12 weeks [11]), lambs (5, 15, 30 and 60 ppm of Cd for 191 days) [12] and chickens (100 ppm Cd for 4 or 9 weeks) [13].

The disruption of iron homeostasis due to long-term exposure to Cd affects human health because iron is an essential metal. According to WHO (2023) [14], anemia was estimated to affect 40% of children aged 6–59 months, 37% of pregnant women and 30% of women aged 15–49 years globally in 2019. Anemia is also the most prevalent in low- and middle-income countries, especially among those living in situations of poverty and social exclusion. It is estimated that nearly a quarter of the world’s population (1.8 billion people) suffered from anemia in 2019. The effects of anemia include a reduction in cognitive and motor development in children, a reduction in fertility and an increase in morbidity and mortality. In addition, anemia can exacerbate the symptoms of other diseases. Iron is absorbed from the duodenum via certain transporters. Orally ingested iron is mainly absorbed from duodenum epithelial cells, and iron homeostasis is regulated by multiple iron-transport-related factors. Two types of iron are in foods: heme and non-heme iron. Heme iron is a complex composed of divalent iron [Fe(II)] and porphyrin, and is abundant in animal sources of food. Non-heme iron is inorganic iron and is found in plant-based foods as trivalent iron [Fe(III)]. Heme iron is more readily absorbed in the duodenum than non-heme iron. On the luminal side of the duodenum, a heme iron transporter, heme carrier protein 1 (HCP1) [15] and a non-heme iron transporter called divalent metal transporter 1 (*Dmt1*) [16] are expressed. *HCP1* is encoded by *Slc46a1* (solute carrier family 46 member 1). *Dmt1* is encoded by *Slc11a2* [solute carrier family 11 (proton-coupled divalent metal ion transporters), member 2]. Heme iron absorbed through *HCP1* is degraded to Fe(II), biliverdin and carbon monoxide by heme oxygenase-1 (HO-1) in enterocytes. Non-heme iron is Fe(III) and is not directly taken up into enterocytes. Fe(III) is reduced to Fe(II) by cytochrome b reductase 1 (Cybrd1) [17], and Fe(II) is taken up into enterocytes via *Dmt1*. Absorbed Fe(II) is released to the vascular side via solute carrier family 40 member 1, also known as efflux transporter ferroportin (Fpn1) [18]. Fe(II) is oxidized to Fe(III) by ceruloplasmin (Cp) and hephaestin (Heph) [19]. The majority of Cp is secreted extracellularly into the blood, while Cp linked to glycosylphosphatidylinositol (GPI) binds to the plasma membrane. In contrast, *Heph* is highly expressed in the basolateral membrane of the small intestine. *Fpn1* expression is post-translationally regulated by hepcidin antimicrobial peptide (Hamp) produced in the liver. When blood iron is in excess, *Hamp* is produced and *Fpn1* is degraded, thereby regulating the iron delivered into the blood [20,21]. Blood iron is transported to various tissues by binding to transferrin.

Our recent study demonstrated that single-dose oral administration of Cd (50 mg Cd/kg body weight) to female mice decreased the serum iron concentration to 54% of the control group and inhibited the expression of iron-transport-related genes in the duodenum 24 h after administration [22]. *Dmt1*, *Fpn1* and *Cybrd1* mRNA levels were markedly higher in the proximal duodenum (2 cm from just below the stomach) than in other parts of the intestine [distal duodenum (2 cm from just below the proximal duodenum), jejunum, ileum, colon, and rectum]. mRNA levels of *Dmt1*, *Fpn1* and *Cybrd1* were 2.5, 4 and 7 times those of the distal duodenum, respectively [22]. Moreover, the expression of iron-transport-related genes was also inhibited in human colon cancer Caco-2 cells, which are a cell model of small intestinal enterocytes, exposed to Cd [22].

The molecular mechanism of decreased iron accumulation in the liver due to long-term exposure to Cd is unknown. Therefore, in this study, we investigated the effect of long-term exposure to Cd not only on the iron concentration in the liver but also on the expression of iron-transport-related genes in the proximal duodenum and liver of mice. 

## 2. Materials and Methods

### 2.1. Animals 

Mouse feed containing 300 ppm Cd was prepared by Oriental Yeast (Tokyo, Japan) with a certified diet (MF, Oriental Yeast) and CdCl_2_ (Fujifilm Wako Pure Chemical., Osaka, Japan). Four-week-old female C57BL/6J mice were purchased from CLEA Japan (Tokyo, Japan) and randomly assigned to control (*n* = 24) or Cd-exposed groups (*n* = 21) (approval protocol code: 14-024). These mice were maintained in the laboratory animal facility of the School of Pharmacy, Aichi Gakuin University, at 23 ± 1 °C, with 55 ± 15% relative humidity. This laboratory animal facility is checked twice a year for contamination by pathogenic microorganisms, and no pathogenic microorganisms were detected during this study period. Mice were acclimated with MF and tap water *ad libitum* for 1 week. All maintenance of mice and experiments with mice were performed in accordance with the guidelines established by the Animal Care and Use Committee of the School of Pharmacy, Aichi Gakuin University.

### 2.2. Animal Treatments

Five-week-old mice were fed MF containing 300 ppm Cd and tap water *ad libitum* for 12, 15, 19 and 21 months. The number of mice in each group was as follows: (1) 12-month control (*n* = 5), (2) 12-month Cd (*n* = 5), (3) 15-month control (*n* = 7), (4) 15-month Cd (*n* = 5), (5) 19-month control (*n* = 6), (6) 19-month Cd (*n* = 5), (7) 21-month control (*n* = 6), (8) 21-month Cd (*n* = 6). Mice were sacrificed under anesthesia and blood (approximately 0.5–1.0 mL per mouse) was collected in a capiject (Terumo, Tokyo, Japan), a container for the collection of a small amount of blood. Serum was separated from the blood in the capiject via centrifugation. The liver and duodenum were removed from mice. The portion just below the stomach (2 cm) was defined as the proximal duodenum. Tissues were snap-frozen and stored at −80 °C until analysis.

### 2.3. Hepatotoxicity 

As indicators of hepatotoxicity, aspartate aminotransferase (AST) and alanine transaminase (ALT) activities in the serum were examined using spectrophotometry, SPOTCHEM EZ SP-4460 (ARKRAY, Kyoto, Japan).

### 2.4. Real-Time RT-PCR Analysis

Initially, 5% (*w*/*v*) homogenates of the proximal duodenum or liver with Lysis Buffer of QuickGene RNA tissue kit (Kurabo, Osaka, Japan) were produced. Subsequently, total RNA was extracted from the 5% (*w*/*v*) homogenate using the QuickGene RNA tissue kit and QuickGene (Kurabo) in accordance with the manufacturer’s protocols. The quantity and purity of RNA were measured using a NanoDrop device (Thermo Fisher Scientific, Waltham, MA, USA). RNA was subjected to a PrimeScript RT reagent kit (Takara Bio, Shiga, Japan) to generate cDNA. Real-time PCR was carried out using SYBR Premix Ex Taq II (Takara Bio) on a Thermal Cycler Dice Real Time System (Takara Bio) in accordance with the manufacturer’s instructions. The sequences of specific primers for mouse genes are listed in Table 1. 

### 2.5. Cd and Fe Concentrations

For metal analysis, serum (20 µL) and liver homogenates (100 µL of 5% (*w*/*v*) homogenates) were digested by wet ashing procedure using nitric acid for metal analysis (60%) (KANTO CHEMICAL, Tokyo, Japan) and hydrogen peroxide (30%) for atomic absorption spectrometry (KANTO CHEMICAL). Serum and liver homogenates added with 2 mL nitric acid were heated using a dry block heater with a Teflon (polytetrafluoroethylene) ball on top of a glass test tube at 80 °C for 30 min, 100 °C for 1 h, 110 °C for 1 h, 120 °C for 2 h and then 140 °C until volatilized without the Teflon ball. Next, 0.5 mL nitric acid and 2 mL hydrogen peroxide were added, followed by heating with a Teflon ball at 80 °C for 1 h, 90 °C for 1 h, 100 °C for 1 h, 110 °C for 1 h, 120 °C for 1 h and then 140 °C until volatilized with 2.5 mL mixture of nitric acid and hydrogen peroxide without the Teflon ball. After sample digestion, Cd and Fe concentrations were measured using the graphite furnace atomic absorption instrument, 280Z AA (Agilent Technologies, Santa Clara, CA, USA). The injection volume was 5 µL. The heating program for Cd was as follows: 85 °C for 5 s, 95 °C for 40 s, 120 °C for 10 s, 250 °C for 6 s with 0.3 L/min argon gas flow, 250 °C for 2 s, 1800 °C for 2.8 s without argon gas and 1800 °C for 2 s with 0.3 L/min argon gas flow. The heating program for Fe was as follows: 85 °C for 5 s, 95 °C for 40 s, 120 °C for 10 s, 700 °C for 6 s with 0.3 L/min argon gas flow, 700 °C for 2 s, 2300 °C for 2.8 s without argon gas and 2300 °C for 2 s with 0.3 L/min argon gas flow. The measurement wavelengths for Cd and Fe were 228.8 nm and 248.3 nm, respectively.

### 2.6. Serum UIBC and TIBC Quantification

The unsaturated iron-binding capacity (UIBC) in the serum was measured using a Microassay UIBC quantification kit (Bathophenanthroline method) (Metallogenics, Chiba, Japan) in accordance with the manufacturer’s instructions. The total iron-binding capacity (TIBC) in the serum was calculated via the summation of the serum iron concentration and serum UIBC.

### 2.7. Statistical Analysis

All values are expressed as the mean ± standard deviation (S.D.). Statistical significance was assessed using analysis of variance and Bonferroni’s multiple *t*-test. *p*-values of less than 0.05 were statistically significant.

## 3. Results

### 3.1. Effect of Long-Term Exposure to Cd on Accumulation of Iron and Cd in the Liver

**Figure 1:**
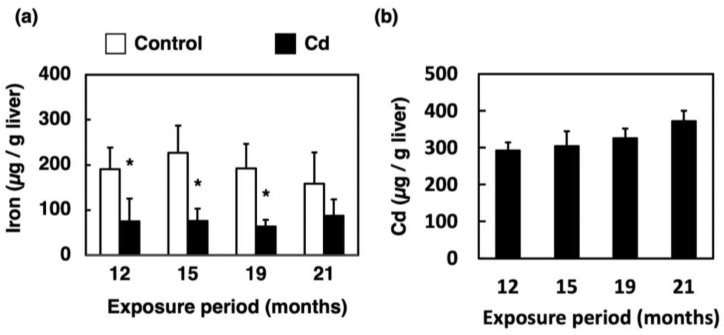
Concentration of iron and Cd in the liver of mice exposed to Cd for a long time. Concentrations of iron (**a**) and Cd (**b**) in the liver were determined after 12, 15, 19 and 21 months of exposure to Cd. Values are means ± S.D. (*n* = 4–6). * Significantly different from the corresponding control, *p* < 0.05. Cd in the liver of the control group was not detected.

As shown in Figure 1a, the total iron concentration in the liver of the mice exposed to Cd for 12 months was significantly decreased compared to that in the control. Additionally, the iron concentration in the liver was reduced by Cd exposure until the 21st month. The Cd concentration in the liver of the mice exposed to Cd for a long time was >300 ppm and slightly increased in an exposure-time-dependent manner (Figure 1b).

### 3.2. Changes in Body Weight and Hepatotoxicity in Mice Exposed to Cd for a Long Time 

**Figure 2:**
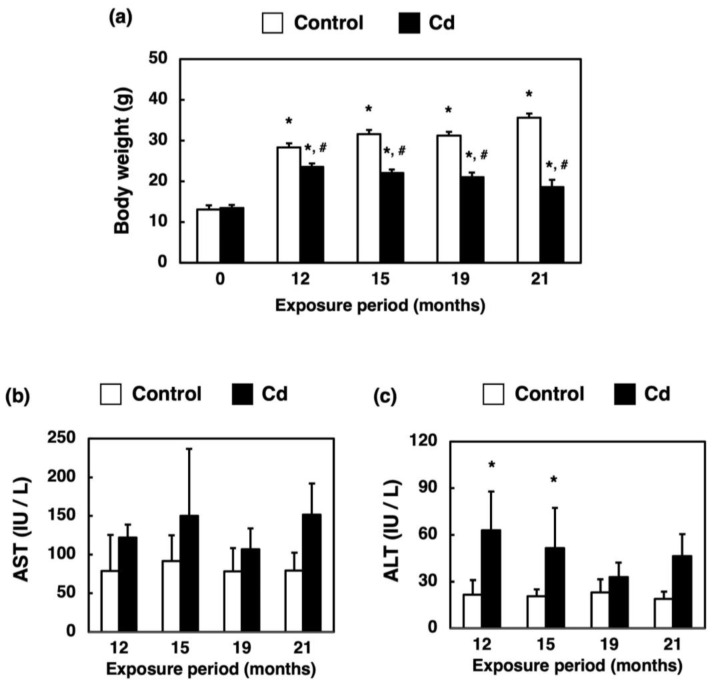
Changes in body weight and activities of AST and ALT in the serum of mice exposed to Cd for a long time. Changes in body weight (**a**) of mice after 12, 15, 19 and 21 months of exposure to Cd. Values are means ± S.D. (*n* = 5–7). * Significantly different from the corresponding 0-month-exposed group, *p* < 0.05. ^#^ Significantly different from corresponding control, *p* < 0.05. Activities of AST (**b**) and ALT (**c**) in the serum were determined after 12, 15, 19 and 21 months of exposure to Cd. Values are means ± S.D. (*n* = 4–7). * Significantly different from the corresponding control, *p* < 0.05.

The body weights of Cd-exposed mice were significantly lower than those of the control group (Figure 2a). AST activity was not altered by long-term exposure to Cd (Figure 2b). ALT activity was increased slightly by exposure to Cd for 12 and 15 months (Figure 2c).

### 3.3. Effect of Long-Term Exposure to Cd on Iron Concentration, UIBC and TIBC in the Serum

**Figure 3:**
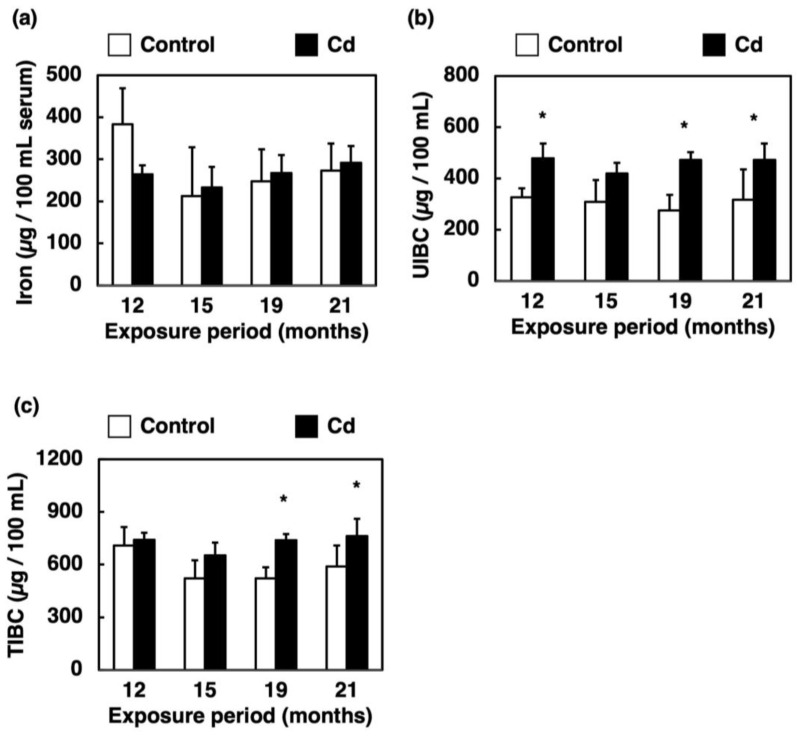
Iron concentration, UIBC and TIBC in the serum of mice exposed to Cd for a long time. Iron concentration (**a**), UIBC (**b**) and TIBC (**c**) in the serum were determined after 12, 15, 19 and 21 months of exposure to Cd. Values are means ± S.D. (*n* = 4–7). * Significantly different from the corresponding control, *p* < 0.05.

Iron in serum is bound to transferrin and transported to the whole body. In a healthy individual, only about one third of the total transferrin in the serum is bound to iron. The amount of iron that can bind to the remaining two thirds of transferrin is called UIBC. The sum of serum iron (iron bound to transferrin) and UIBC, i.e., the amount of iron that can bind to the total transferrin in the serum, is called TIBC [23]. The iron concentration in the serum was not changed by long-term exposure to Cd (Figure 3a). UIBC was significantly increased by long-term exposure to Cd except for the 15-month exposure (Figure 3b). TIBC was significantly increased by 19 and 21 months of exposure to Cd (Figure 3c).

### 3.4. Effect of Long-Term Exposure to Cd on the Expression of Genes Involved in Absorption of Heme Iron in the Proximal Duodenum 

**Figure 4:**
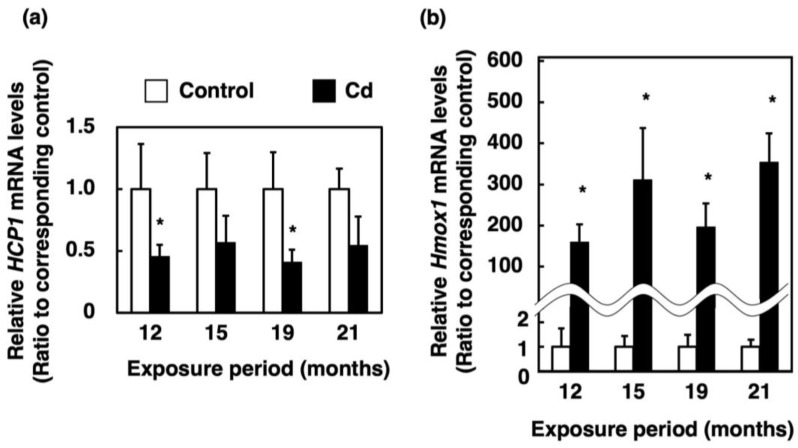
Expression of genes involved in heme iron absorption in the proximal duodenum of mice exposed to Cd for a long time. mRNA levels of *HCP1* (**a**) and *Hmox1* (**b**) in the proximal duodenum were determined after 12, 15, 19 and 21 months of exposure to Cd. mRNA levels were normalized to *Actb* mRNA expression. Values are means ± S.D. (*n* = 4–7). * Significantly different from the corresponding control, *p* < 0.05.

To elucidate the cause of loss of stored iron in the liver induced by Cd, we measured the expression of heme iron influx transporters in the proximal duodenum. The mRNA level of *HCP1*, which encodes a heme iron influx transporter in the Cd-exposed group, was approximately half of that in the control, which was significantly decreased at 12 and 19 months of exposure to Cd (Figure 4a). The *Hmox1* is the gene encoding HO-1. The *Hmox1* mRNA level was drastically increased by Cd (Figure 4b).

### 3.5. Effect of Long-Term Exposure to Cd on the Expression of Genes Involved in Absorption of Non-Heme Iron in the Proximal Duodenum 

**Figure 5:**
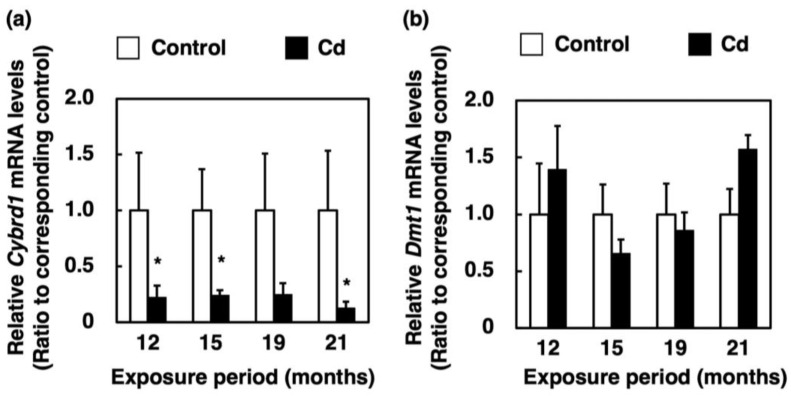
Expression of genes involved in non-heme iron absorption in the proximal duodenum of mice exposed to Cd for a long time. mRNA levels of *Cybrd1* (**a**) and *Dmt1* (**b**) in the proximal duodenum were determined after 12, 15, 19 and 21 months of exposure to Cd. mRNA levels were normalized to *Actb* mRNA expression. Values are means ± S.D. (*n* = 4–6). * Significantly different from the corresponding control, *p* < 0.05.

To elucidate the cause of loss of stored iron in the liver induced by Cd, we measured the expression of non-heme iron influx transporters in the proximal duodenum. The *Cybrd1* mRNA level was dramatically suppressed by long-term exposure to Cd (Figure 5a). However, the mRNA level of *Dmt1*, which encodes a non-heme iron influx transporter, was not affected by Cd (Figure 5b).

### 3.6. Effect of Long-Term Exposure to Cd on the Expression of Genes Involved in the Efflux of Iron from the Proximal Duodenum Enterocytes into Blood Vessels

**Figure 6:**
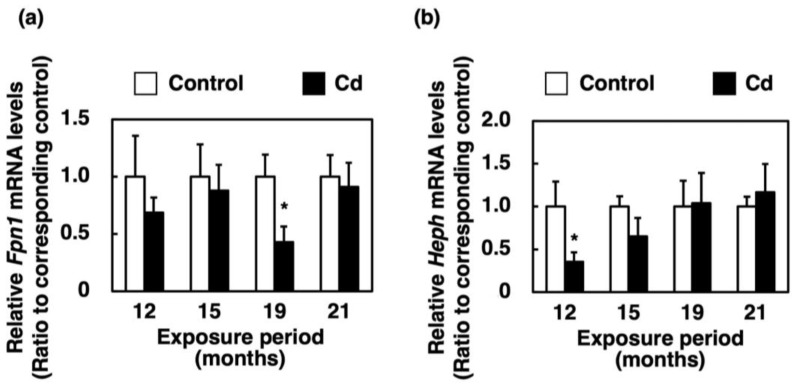
Expression of genes involved in iron efflux from the proximal duodenum enterocytes into blood vessels in mice exposed to Cd for a long time. mRNA levels of *Fpn1* (**a**) and *Heph* (**b**) in the proximal duodenum were determined after 12, 15, 19 and 21 months of exposure to Cd. mRNA levels were normalized to *Actb* mRNA expression. Values are means ± S.D. (*n* = 3–7). * Significantly different from the corresponding control, *p* < 0.05.

*Fpn1* and *Heph* mRNA levels were generally not altered by long-term exposure to Cd, but they were significantly reduced at some time points (Figure 6a,b).

### 3.7. Effect of Long-Term Exposure to Cd on Gene Expression of Hamp and Fpn1 in the Liver 

**Figure 7:**
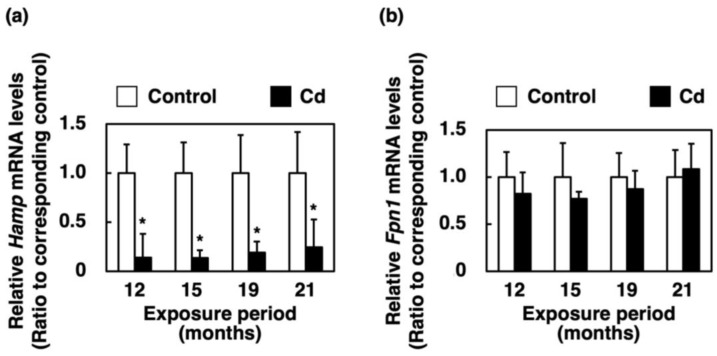
Gene expression of *Hamp* and *Fpn1* in the liver of mice exposed to Cd for a long time. mRNA levels of *Hamp* (**a**) and *Fpn1* (**b**) in the liver were determined after 12, 15 19 and 21 months of exposure to Cd. The mRNA level was normalized to *Actb* mRNA expression. Values are means ± S.D. (*n* = 3–7). * Significantly different from the corresponding control, *p* < 0.05.

The mRNA level of Hamp, which restricts *Fpn1* expression, in the liver of mice was significantly decreased in all of the Cd exposure period groups (Figure 7a). The *Fpn1* mRNA level in the liver of mice exposed to Cd for a long time was not changed (Figure 7b).

## 4. Discussion

As shown in Figure 1b, hepatic Cd concentrations increased slightly, but only in a period-dependent manner. Thijssen et al. [24] reported that in mice exposed to 100 mg Cd/L drinking water for 23 weeks, renal Cd concentrations increased in a period-independent manner while hepatic Cd concentration peaked at 16 weeks of Cd exposure and began to decrease at 23 weeks. Although Cd accumulation in the liver increases in the early phase of exposure, Cd concentration in the liver eventually becomes stabilized due to the transportation of Cd as Cd-metallothionein (CdMT) from the liver to the kidney. Therefore, the present study suggests that hepatic Cd is maintained at the same concentration during long-term Cd exposure. In this study, significant increases in ALT were observed in the Cd 12- and 15-month exposure groups, but the mean values were about 60 IU/L (Figure 2c). These were not high enough to determine the appearance of hepatotoxicity. In addition, no variation in AST values due to Cd exposure was observed (Figure 2b). From these results, it can be concluded that no noteworthy hepatotoxicity appeared throughout the Cd exposure period. Therefore, long-term exposure to Cd showed little apparent hepatotoxicity.

**Figure 8:**
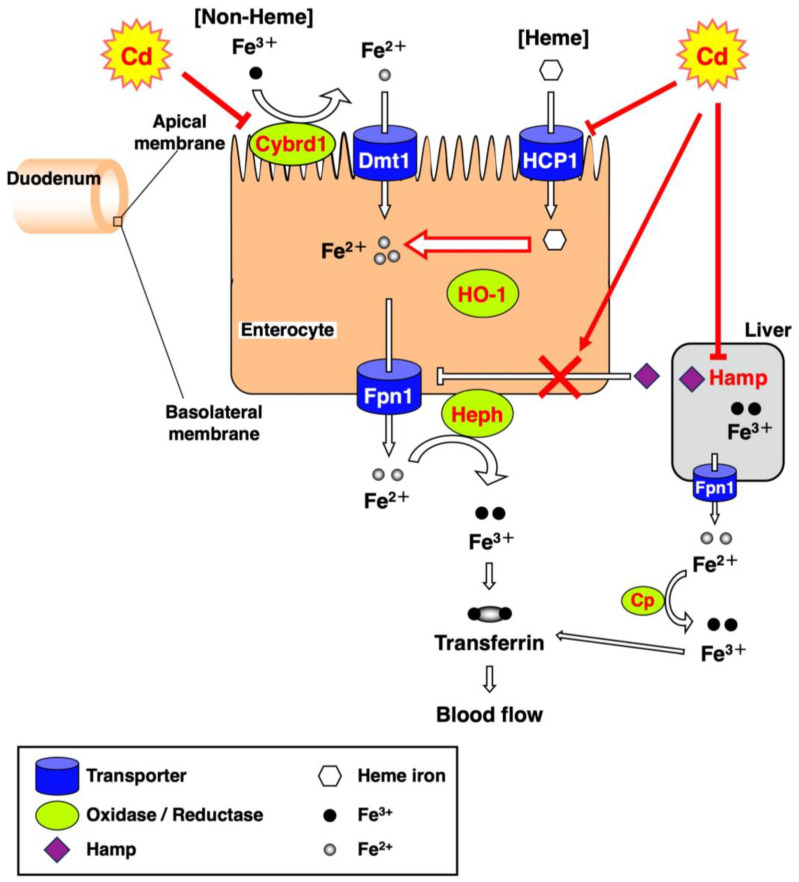
Proposed scheme which rationalizes the long-term exposure of mice to Cd on the metabolism of Fe.

Our previous study showed that the serum iron concentration and TIBC of mice were significantly decreased at 24 h after a single administration of Cd [22]. In the present study, on the other hand, the serum iron levels in mice exposed to Cd for a long time remained at the control levels (Figure 3a), which did not induce an iron deficiency. However, iron accumulation in the liver was markedly decreased (Figure 1a) and UIBC was significantly elevated after 12 months of exposure to Cd (Figure 3b). Moreover, Figure 3c indicates a significant increase in the TIBC of mice exposed to Cd for 19 and 21 months. As shown in Figure 3a, there was no "apparent" change in serum iron levels by the long-term exposure to Cd in this experiment. However, the iron concentration in the liver was significantly decreased (Figure 1a) and the UIBC and TIBC levels were significantly increased (Figure 3b,c) via long-term exposure to Cd. These findings imply that the biological response to ameliorate the iron deficiency state may occur by increasing the amount of transferrin that can bind to iron in the serum. Therefore, these results suggested that the mice exposed to Cd were severely iron-deficient. Excess iron absorbed is stored in the liver as ferritin and is released into the blood through *Fpn1* expressed in the liver when iron deficiency occurs [25]. *Fpn1* is highly expressed in duodenal enterocytes, liver Kupffer cells, splenic red pulp macrophages, periportal hepatocytes and the placental syncytiotrophoblast [25]. Subsequently, the Fe(II) excreted from the liver is oxidized to Fe(III) mainly by Cp, and then binds to transferrin (Figure 8). Therefore, long-term Cd exposure may consume iron in the liver while maintaining iron concentrations in the serum.

In the present study, the expression of *HCP1* and *Cybrd1* in the proximal duodenum where iron from food is absorbed, and the expression of *Hamp* in the liver, were significantly suppressed in female C57BL/6J mice orally administered with 300 ppm Cd for more than 1 year (Figure 4a, Figure 5a and Figure 7a). The gene expression of HCP1, a heme iron transporter, in the Cd exposed group was approximately half of the control group and significantly decreased after 12 and 19 months of exposure (Figure 4a). On the other hand, the gene expression of *Dmt1*, which is a non-heme iron transporter, did not change after exposure to Cd, compared to the control (Figure 5b). However, *Cybrd1* gene expression was significantly reduced throughout the periods of exposure to Cd (Figure 5a). *Cybrd1* is responsible for the reduction of Fe(III) to Fe(II) and is required for non-heme iron to be taken up into cells through *Dmt1*. These results suggest, therefore, that the uptake of both heme- and non-heme iron into enterocytes was diminished by Cd. It is well known that Cd readily induces HO-1 expression [26]. In the present study, long-term exposure to Cd also markedly elevated *Hmox1* expression in the enterocytes (Figure 4b). Since the expression level of *HCP1* was reduced by Cd, heme iron uptake into enterocytes was expected to be reduced, but the uptake of heme iron is expected to be rapidly degraded to Fe(II) by HO-1, whose expression was increased in the enterocytes. Iron taken up into enterocytes is released into blood via Fpn1. *Heph* and Cp are enzymes that oxidize Fe(II) released into the vascular side to Fe(III). It is known that *Heph* is a homolog of Cp with 50% homology at the amino acid level [19]. As shown in the review by Helman et al. [27], Cp particularly works in the liver and certain cell types in the brain and pancreas [28,29,30], whereas the major oxidative enzyme in the small intestine is *Heph* [31]. Therefore, we investigated the effect of long-term exposure to Cd on *Heph* in the duodenum of mice. Cd did not induce a change in the expression of *Fpn1* and *Heph* genes, which are involved in the release of iron from the duodenum into blood vessels (Figure 6a,b). Furthermore, gene expression of the peptide hormone Hamp, which degrades Fpn1, in the liver remained significantly reduced throughout the Cd exposure period (Figure 7a). Thus, it is likely that the reduced expression of *HCP1* and *Cybrd1* in the Cd-exposed group reduced the amount of iron taken up by intestinal cells, but that the small amount of iron taken up was released into the bloodstream as normally as in the control group. Moreover, the mRNA level of *Fpn1* in the liver was not altered by Cd exposure (Figure 7b). *Hamp* produced in the liver is also involved in the degradation of *Fpn1* in the liver. It is reasonable that the *Fpn1* mRNA level in the liver was not altered by Cd exposure since *Hamp* production was significantly suppressed by Cd exposure (Figure 7a). However, the iron concentration in the liver of Cd-exposed mice was about half of the control. Therefore, it was indicated that Cd-exposed mice may not have been able to accumulate as much iron in the liver as the control group because iron absorption was inhibited, and iron was released from the liver into the blood.

Ohta and Ohba [32] reported that the *Dmt1* mRNA level in the duodenum of rats was significantly increased by oral exposure to Cd for 5 weeks. Similarly, orally ingested Cd increased the *Dmt1* mRNA level in the duodenum of pregnant rats [33]. However, in our previous study, it was reported that the expression of *HCP1*, *Dmt1*, *Cybrd1*, *Fpn1* and *Heph* in the duodenum of mice was significantly reduced at 24 h after a single oral administration of Cd [22]. Moreover, the gene expression of *Hamp* in the liver of mice in that study was significantly reduced at 24 h after exposure to Cd. Short-term Cd exposure sensitively reduced the expression of almost all iron-transport-related factors. However, long-term Cd exposure resulted in an expression comparable to control levels except for *HCP1* and *Cybrd1*, due to homeostasis that maintains normal in vivo levels of iron. Thus, it is suggested that *HCP1* and *Cybrd1* may be key factors in Cd-induced iron deficiency.

In conclusion, the present study found that long-term exposure to Cd decreases iron accumulation in the liver, and suppresses the expression of *HCP1* and *Cybrd1*, which are associated with iron absorption into duodenal epithelial cells, indicating that iron absorption may be inhibited (Figure 8). Moreover, these results suggest that iron stored in the liver is released into serum, leading to a potential iron deficiency and suggesting the need for aggressive iron supplementation to counteract the chronic toxicity of Cd in mammals. Anemia is a health problem of global concern. Our findings suggest that long-term exposure to Cd may exacerbate iron deficiency. Therefore, it is proposed that populations at high risk for anemia have more of a reduced Cd intake from their diet and luxury items such as cigarettes, and take iron supplementation in order to prevent anemia and various health problems caused by anemia.

## Figures and Tables

**Table 1:** Sequences of mouse primers (5′–3′).

Gene Names	Forward	Reverse	Product Size (bp)
*Slc46a1* (*HCP1*)	ATCTACCCGGCCACTCTGAA	GGAACTCCGGGTGTGGATTA	121
*Hmox1*	ACCTTCCCGAACATCGACAG	GGAAGGCGGTCTTAGCCTCT	121
*Slc11a2* (*Dmt1*)	GGCTTTCTTATGAGCATTGCCTA	GGAGCACCCAGAGCAGCTTA	97
*Cybrd1*	GCAGCGGGCTCGAGTTTA	TTCCAGGTCCATGGCAGTCT	103
*Slc40a1* (*Fpn1*)	CTGTCGGCCAGATTATGACA	GAGCAGGGGTCTTCTGGTAA	126
*Heph*	TTGTCTCATGAAGAACATTTACAGCAC	CATATGGCAATCAAAGCAGAAGA	161
*Hamp*	GGCAGACATTGCGATACCAA	TGGCTCTAGGCTATGTTTTGCA	128
*Actin, beta* (*Actb*)	CCTAAGGCCAACCGTGAAAA	AGGCATACAGGGACAGCACA	100

## Data Availability

All data is contained within this manuscript.
